# A Case of Asymptomatic Massive Inguinoscrotal Bladder in Acute Renal Failure

**DOI:** 10.7759/cureus.43139

**Published:** 2023-08-08

**Authors:** Annalee Mora, Opeyemi Oyenusi, Amirali Ghavamrezaii, Safwan Mohiuddin, Nikolay Mitzov

**Affiliations:** 1 Internal Medicine, HCA Florida Healthcare Oak Hill Hospital, Brooksville, USA

**Keywords:** hydronephrosis, pulmonary edema, inguinal hernia, acute renal failure, inguinoscrotal bladder

## Abstract

Inguinal hernia is a common condition that typically affects males in the age group of 50-70 years. While often asymptomatic or mildly symptomatic, complications such as urinary bladder herniation and obstructive uropathy can occur if left untreated. We present a unique case of a 60-year-old man with a body mass index of 37 kg/m^2^ with a 20-year history of untreated bilateral inguinal hernias. His condition progressed to a complicated right inguinoscrotal hernia involving the bladder, leading to obstructive uropathy, acute renal failure, and pulmonary edema. Diagnostic imaging revealed hydronephrosis and obstruction of the distal right ureter, necessitating several procedures, including diuretic therapy, a nephrostogram, a nephrostomy, and ultimately hemodialysis due to persistent renal failure. Surgical management was achieved through an emergent robotic-assisted repair of the right inguinal hernia using resorbable mesh while repairing the left hernia was delayed to mitigate potential risks. This case illustrates the severe complications that can arise from a longstanding untreated inguinal hernia, highlighting the importance of routine monitoring and early intervention. It also emphasizes the diagnostic role of different imaging modalities and immediate pharmacological and surgical intervention in managing such complications. Despite the commonality of inguinal hernia, a lack of timely treatment can lead to life-threatening conditions, necessitating a comprehensive approach to management to improve patient outcomes.

## Introduction

Levine first reported an inguinoscrotal herniation of the bladder, otherwise known as a massive scrotal cystocele, in 1951 [1]. This condition is uncommon and is often diagnosed intraoperatively [2]. Clinical symptoms include "double urination" [3], which might be accompanied by urinary urgency, frequency, or retention but is generally asymptomatic. Prolonged herniation can result in serious complications such as sepsis, obstructive uropathy, hydronephrosis, renal failure, and even end-stage renal disease. These complications can affect the lungs and the heart in severe cases, with the most extreme outcomes leading to bladder ischemia or gangrene [3]. Management options range from conservative care to surgical intervention via open or laparoscopic methods.

We report a case of an asymptomatic inguinoscrotal bladder persisting for 20 years, which led to acute renal failure and pulmonary edema due to obstruction and hydronephrosis. This report underscores the importance of an interdisciplinary team approach in providing urgent intervention to enhance renal function, acknowledging that full recovery remains a challenge.

## Case presentation

A 60-year-old male with a 20-year history of bilateral inguinal hernia visited the emergency department presenting with dyspnea on exertion, headache, and chest pressure that persisted for two weeks. He was previously assessed at an urgent care facility for right knee pain, where his systolic blood pressure was above 200 mmHg. The urgent care center referred him to the emergency department for further evaluation and management. He had been taking ibuprofen to alleviate knee pain with partial success. Two months prior, he reported progressively worsening lower extremity edema, exertional dyspnea, and orthopnea. Furthermore, he noticed his bilateral inguinal hernia had become more burdensome over the past two years. The patient did not report chest pain, fever or chills, dizziness, headaches, abdominal pain, nausea, vomiting, dysuria, or urinary urgency or frequency.

**Figure 1:**
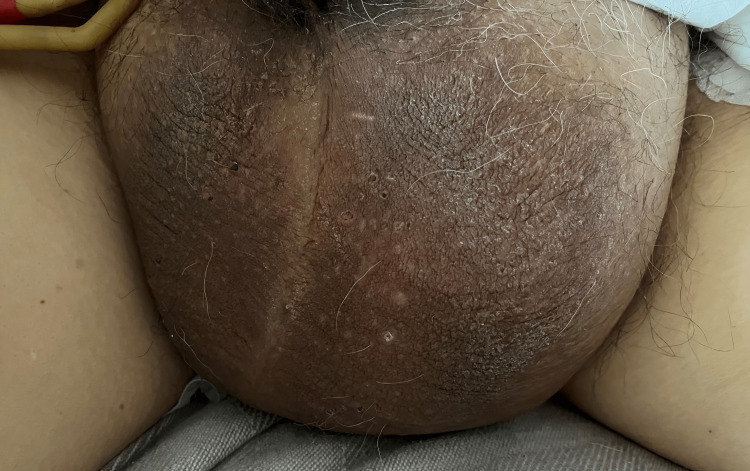
Massive inguinal hernia

**Figure 2:**
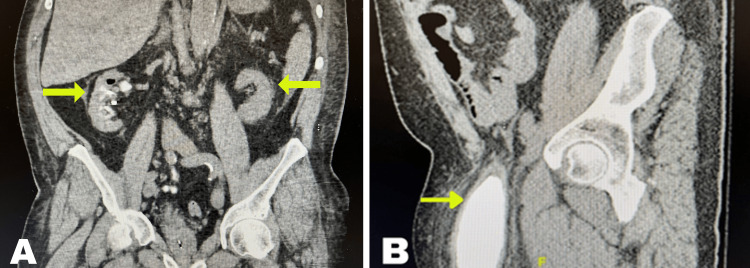
Abdominal CT scan showing: (A) bilateral atrophic kidney; (B) anterior aspect of the urinary bladder in the right inguinal hernia (coronal view) CT, computed tomography

The patient's blood pressure was 199/106 mmHg, heart rate was 85 beats per minute, temperature 36.5°C, respiratory rate 18, and oxygen saturation was 97% on room air. His body mass index (BMI) was 37 kg/m^2^. He was awake, alert, oriented, and in moderate distress. His head was atraumatic and normocephalic without visual field defects. Auscultation detected a regular systolic murmur and bibasilar crackles. The patient exhibited significant, nontender, bilateral massive inguinal hernias (Figure 1) and 2+ pitting edema in his lower extremities without cyanosis, lesions, and preserved bilateral sensory function.

Laboratory results showed low hemoglobin and hematocrit, slightly low sodium, and elevated levels of potassium, phosphorus, serum urea nitrogen, creatinine, troponins, brain natriuretic peptide, and lactate dehydrogenase (Table 1). The patient's glomerular filtration rate (GFR) was significantly decreased, and proteinuria was observed. An arterial blood gas revealed metabolic acidosis with respiratory compensation.

**Table 1:** Laboratory workup results

Parameter	Values	Reference Values
Hemoglobin	8.6 g/dL	13.7-15 g/dL
Hematocrit	26.1 %	40-51 %
Sodium	134 mmol/L	136-145 mmol/L
Potassium	5.2 mmol/L	3.5-5.1 mmol/L
Phosphorus	10.0 mg/dL	2.5-4.9 mg/dL
Serum urea nitrogen	142 mg/dL	7.0-18.0 mg/dL
Creatinine	16.3 mg/dL	0.7-1.3 mg/dL
Glomerular filtration rate	3.04 ml/min	> 60 ml/min
Troponin	2169 ng/L	< 78 ng/L
Pro-brain natriuretic peptide	57234 pg/ml	5.0-125 pg/ml
Lactate dehydrogenase	354 units/L	100-240 units/L

**Figure 3:**
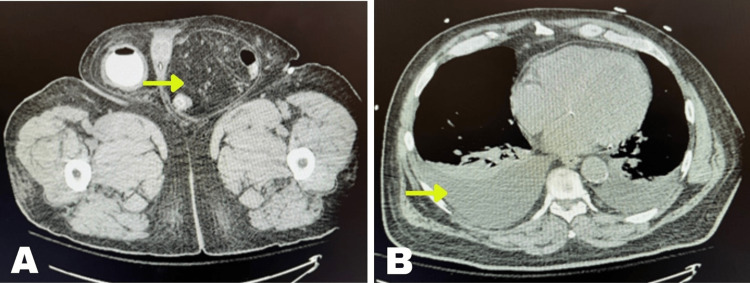
(A) Abdominal CT displaying a massive left inguinal hernia; (B) Chest CT scan featuring pulmonary edema CT, computed tomography

His electrocardiogram showed QT prolongation without ST-segment elevation. Echocardiography revealed an ejection fraction of 59% with mild to moderate valvular disease. Bilateral lower extremity ultrasound showed no evidence of deep vein thrombosis. Abdominal and pelvic computed tomography (CT) scans with contrast revealed a large right inguinal hernia containing the anterior aspect of the urinary bladder extending below the field of view (Figure 2). This hernia was causing distal right ureter obstruction and cortical thinning. There was also a substantial left inguinal hernia (Figure 3A), apparently containing the distal sigmoid colon and fat. Subsequent renal ultrasound disclosed significant right-sided hydronephrosis. A chest X-ray and CT scan confirmed pulmonary edema and cardiomegaly (Figure 3B).

The patient developed acute hypoxic respiratory failure due to pulmonary edema, necessitating noninvasive ventilation and admission to the intensive care unit. He was initiated on a nitroglycerine drip, intravenous furosemide, and continuous bilevel-positive airway pressure. His hemoglobin dropped the following day to 6.8 g/dL from 8.6 g/dL. However, it was improved to 7.9 g/dL after receiving one unit of packed red blood cells.

A nephrologist and urologist were involved in the patient's management. As his kidney function deteriorated and urine output decreased, a nephrostogram and nephrostomy were performed. Despite increased urine output following these interventions, the patient's longstanding obstructive uropathy, right renal cortical thinning, and atrophic left kidney indicated that surgical intervention was unlikely to enhance renal function long-term. The decision was made to start hemodialysis. The patient's blood pressure improved, and serum urea nitrogen and creatinine levels gradually decreased, yet his GFR remained significantly low at 7.12 ml/minute.

Given the immediate concerns regarding obstructive uropathy, surgical repair of the right inguinal hernia containing the urinary bladder was undertaken, repositioning the bladder within the abdominal cavity. Repair of the left inguinal hernia was delayed due to concerns about potential diaphragmatic compression and increased abdominal pressure. The patient continues to undergo hemodialysis.

## Discussion

The incidence of inguinal hernia increases with age, typically affecting males aged 50-70 years [4]. Although it is one of the most common surgical procedures [5], an inguinal hernia can remain asymptomatic or only mildly symptomatic for years. In the current patient, factors such as a BMI of 37 kg/m^2^ and advancing age likely exacerbated the development of this condition due to abdominal wall weakness and degeneration of muscle fibers. Risk factors for inguinal hernia include obesity, increased abdominal pressure, and age [1]. If not repaired promptly, chronic inguinal hernias can lead to complications, including herniation of the urinary bladder, a rarity found in 4% of inguinal hernias [5]. The development of bladder herniation can result from a gradual weakening of supporting structures, bladder outlet obstruction, and chronic bladder overdistension [6].

The patient's longstanding bilateral inguinal hernia of 20 years resulted in complications such as obstructive uropathy and hydronephrosis, eventually causing renal failure and pulmonary edema. This increased pressure proximal to the obstruction led to a decrease in the GFR. Obstructive uropathy also resulted in electrolyte and metabolic abnormalities, as observed in this patient. While renal failure is usually rare, prolonged neglect can lead to ureteric involvement. Persistent obstruction for over six weeks can contribute to irreversible chronic renal disease development [7]. An enlarging inguinal hernia can also limit mobility, causing difficulty in walking [8], and may result in back, hip, and knee pain due to increased heaviness, as likely seen in the patient's bilateral knee pain.

Although inguinal hernia is a clinical diagnosis and imaging is not routinely recommended, imaging studies may be required for recurrent lower urinary tract infections, decreasing urinary output, and diagnostic uncertainty [1,5]. The patient's complex presentation required various tests to evaluate the extent of the pathology. A CT scan, the primary imaging modality, can help assess hernia size, position, surrounding abnormalities, and potential surgical management [5,9]. The present patient's CT scan was highly sensitive, confirming complications such as hydronephrosis. It revealed obstruction of the distal right ureter and cortical thinning due to longstanding kidney disease. CT scans are also recommended in obese individuals and men over the age of 50 [10]. An ultrasound, which our patient underwent, is another suitable method for detecting hydronephrosis. Although voiding cystography is the optimal imaging technique for evaluating urethral and bladder abnormalities in the scrotum [5], it was unnecessary in this case since the CT scan already confirmed the pathology.

The patient's renal impairment, increased blood volume, and reduced renal clearance resulted in elevated B-type natriuretic peptide and troponin levels. As a result, diuretic therapy was initiated to alleviate symptoms of pulmonary edema, acute renal failure, and suspected heart failure. However, due to the lack of the desired beneficial effect, immediate nephrostogram and nephrostomy procedures were performed to decompress the obstructed distal right ureter. These life-saving procedures improved the patient's urine output. Despite gradual improvement in renal function from admission, hemodialysis was eventually necessary due to persisting renal failure, likely resulting from longstanding obstructive uropathy. The patient's kidney function did not fully recover following the nephrostomy procedure, suggesting progression to end-stage renal disease and necessitating maintenance hemodialysis. An emergent robotic-assisted repair of the right inguinal hernia using resorbable mesh was undertaken, with the bladder repositioned into the abdominal cavity. Given that the patient had a bilateral massive inguinal hernia, simultaneous repair of the left hernia-requiring a return of much of the colon to the abdominal cavity could have increased the risk of diaphragmatic compression and elevated abdominal pressure. Thus, a delay in the repair of the left inguinal hernia was deemed reasonable.

## Conclusions

We described a unique case of a male with a progressive, longstanding history of bilateral inguinal hernia presenting with complications of his right inguinoscrotal bladder. His volume overload symptoms were due to obstructive uropathy, resulting in acute renal failure necessitating hemodialysis. This case underscores the risk factors contributing to his complications and the pathological processes causing his clinical symptoms. We have also highlighted the various imaging modalities that can assist in assessing the severity of the herniation and identifying associated complications. Upon the emergence of such complications, immediate pharmacological intervention and surgical procedures should be implemented to alleviate symptoms, as was done successfully in our patient. Inguinal hernia, often asymptomatic in many patients, can pose life-threatening challenges if not routinely monitored, particularly when irreversible complications have already occurred.
